# Assessment of bone marrow infiltration in B-cell non-Hodgkin's lymphoma (NHL).

**DOI:** 10.1038/bjc.1989.161

**Published:** 1989-05

**Authors:** E. L. Dorey, S. V. Outram, A. Holder, A. Z. Rohatiner, F. E. Cotter, M. Deane, A. G. Stansfeld, T. A. Lister, M. A. Horton

**Affiliations:** Imperial Cancer Research Fund, Department of Medical Oncology, London, UK.


*Br. J. Cancer* (1989), **59**, 772–774 © The Macmillan Press Ltd., 1989

SHORT COMMUNICATION

### Assessment of bone marrow infiltration in B-cell non-Hodgkin's lymphoma (NHL)

E.L. Dorey[1], S.V. Outram[1], A. Holder[2], A.Z.S. Rohatiner[1], F.E. Cotter[1], M. Deane[3], A.G. Stansfeld[4], T.A. Lister[1] & M.A. Horton[2,3]

[1]*Imperial Cancer Research Fund, Department of Medical Oncology;* [2]*Imperial Cancer Research Fund, Haemopoiesis Research Group;* [3]*Department of Haematology, St Bartholomew's Hospital; and* [4]*Department of Histopathology, St Bartholomew's Hospital, West Smithfield, London EC1A 7BE, UK.*

A study is currently in progress at St Bartholomew's Hospital in which patients with B-cell lymphoma receive cyclophosphamide and total body irradiation supported by autologous bone marrow transplantation. The marrow is being treated *in vitro* with the monoclonal antibody anti-B1 (Coulter Immunology) and baby rabbit complement (Pel-Freez) before re-infusion (Rohatiner *et al.*, 1986).

Anti-B1 is an antibody that defines a 35 kDa B-cell differentiation antigen (CD20) which is expressed on more than 95% of normal B-cells isolated from peripheral blood or lymphoid tissue (Stashenko *et al.*, 1980) and the majority of tumour cells from patients with B-cell NHL (Anderson *et al.*, 1984). It has been previously reported that normal bone marrow may contain up to 5% CD20 positive lymphocytes (Stashenko *et al.*, 1981) and that this figure is compatible with absence of bone marrow infiltration by morphological criteria (Nadler *et al.*, 1984). However, *in vitro* culture studies suggest that morphologically normal bone marrow may contain occult lymphoma cells: in a study of Burkitt lymphoma (Benjamin *et al.*, 1984) 17% of morphologically normal bone marrows cultured *in vitro* gave rise to tumour cell lines or cells containing the 8:14 translocation.

A number of patients with morphologically normal bone marrow have been found, on cell surface phenotyping in this laboratory, to have levels of CD20 positive lymphocytes greater than 5%. This observation prompted the present study, the objective of which was first to establish the normal range of CD20 positive lymphocytes in bone marrow and subsequently, to compare the levels of CD20 positive cells in marrow from patients with NHL, before and after therapy. The levels of other immunological markers were also evaluated to estimate the significance of possible artifactual effects which may result from therapy or peripheral blood contamination.

Bone marrow samples from 20 normal subjects (12 men, 8 women, age range 19–43 years, median 31) and 85 patients with NHL (49 men, 36 women; age range 12–80 years, median 53) were studied. The morphological diagnoses are shown in Table I. Patients were categorised into three groups according to bone marrow aspirate and trephine morphology using standard haematological criteria applied in this laboratory, i.e. those in whom the bone marrow was uninvolved before or after therapy and those with bone marrow infiltration.

**Table I** Morphological diagnoses

| *Histology* | *No. of cases* |
|---|---|
| Low grade | 60 |
| High grade | 25 |
| Total | 85 |

Correspondence: E.L. Dorey.
Received 18 April 1988, and in revised form, 2 December 1988.

Bone marrow was aspirated from the posterior iliac crest and added to 10 ml of TC199 medium and preservative-free heparin (100 units). A trephine biopsy was performed on all patients. The marrow was layered on to lymphoprep (Nyeegard), density 1.077, and centrifuged at 1,300 r.p.m. for 25 min at 18°C. The mononuclear cell interface was harvested and washed twice in phosphate buffered saline (PBS) containing 5% calf serum and 0.05% sodium azide. Phenotyping was performed by a modification of standard methods: $10^6$ cells in 20 $\mu$l were incubated with 20 $\mu$l of the test antibody at 2 × working concentration in a 96-well plate (Limbro, Flow) at 4°C for 30 min. The cells were then washed and incubated with 20 $\mu$l of the second antibody, goat anti-mouse FITC (Coulter), for a further 30 min at 4°C. Finally, the cells were washed three times in 5% calf serum in PBS and resuspended in a final volume of 100 $\mu$l of isoton. Each sample was analysed on the day of collection on an Epics C cell sorter (Coulter Electronics) and the percentage of positive cells determined after calibration of the instrument with negative and positive controls.

The following antibodies were used (Table II): anti-B1 (CD20), anti-B4 (CD19), J5 (CD10), CA2 (anti-DR), anti-T3 (CD3) and WT1 (CD7) together with a positive control 2D1 (Leucocyte Common Antigen, CD45) and a negative control, 20 $\mu$l of 5% calf serum in PBS.

The mean and standard deviations were calculated using the Mann–Whitney test. The negative background on the Epics C ranged from 0.1 to 4.0% (the average being 1%, with only one patient having a background value of 4%). The Mann–Whitney test was also used to determine whether there was any significant difference between the results for previously untreated patients and those who had received chemotherapy and between those with involved and uninvolved marrows.

The results are shown in Table III and illustrated in Figure 1. The data have been pooled for all histological subtypes of NHL. The number of CD20 positive lymphocytes was significantly higher in morphologically involved marrow than in marrow from normal subjects (mean values 46% *vs* 8%) (Table III and Figure 1). There was also a significant difference between uninvolved and morphologically involved marrow (Table IV and Figure 1), the CD20 value for involved marrow always being greater than

**Table II** Panel of antibodies used in the study

| *Antibody* | *Antigen (CD number)* | *Source* | *Target* |
|---|---|---|---|
| Anti-B1 | 20 | Coulter | Mature B-cells |
| Anti-B4 | 19 | Coulter | Early B-cell marker |
| J5 | 10 | Coulter | Common ALL antigen |
| CA2 | HLA-DR | W. Bodmer | HLA-DR antigen |
| Anti-T3 | 3 | Coulter | Mature T-cells |
| WT1 | 7 | M. Greaves | Most peripheral T-cells and thymocytes |

**Table III** Immunophenotype (mean±s.e.m.) of bone marrow from normal subjects and patients with NHL

| | % antibody positive cells | | | | | |
|---|---|---|---|---|---|---|
| | *B1 (CD20)* | *B4 (CD19)* | *J5 (CD10)* | *CA2 (HLA-DR)* | *T3 (CD3)* | *WT1 (CD7)* |
| Normal subjects (*n*=20) | 8.2±1.8 | 9.5±2.1 | 9.0±2.0 | 22.7±5.0 | 19.8±4.4 | 12.8±2.8 |
| Uninvolved at presentation (*n*=25) | 5.8±1.2 | 4.4±1.0 | 6.2±1.5 | 27.4±6.1 | 16.3±3.6 | 15.5±3.3 |
| Uninvolved post-treatment (*n*=40) | 5.8±0.9 | 5.1±0.9 | 5.5±1.0 | 20.2±3.3 | 15.1±2.7 | 12.9±2.1 |
| Involved at presentation (*n*=20) | 46.2±10.3 | 42.0±9.6 | 18.8±4.7 | 49.7±12.2 | 14.7±3.5 | 9.1±2.3 |

13% (Figure 1). The same overall pattern applies to antibodies to CD19 and CA2 which are also expressed by B-lymphocytes (Table III).

**Table IV** Statistical comparison between uninvolved and involved bone marrow at presentation

| | |
|---|---|
| Anti-B1 | *P*<0.00001 |
| Anti-B4 | *P*<0.00001 |
| J5 | n.s. |
| CA2 | *P*<0.0002 |
| Anti-T3 | n.s. |
| WT1 | n.s. |

**Figure 1** Levels of CD20 positive lymphocytes in: (**a**) patients with involved marrow; (**b**) normal subjects; (**c**) patients with no evidence of bone marrow infiltration at presentation; and (**d**) patients with no evidence of bone marrow infiltration after therapy.

In contrast, there was no significant difference between the CD20 levels in patients with morphologically normal bone marrow at presentation and after therapy once complete remission had been achieved (Table III). The mean percentage of CD20 positive cells in uninvolved bone marrow was 5.8 both at presentation and after therapy, the median values being 5.0 and 4.0, respectively. There was also no significant difference between the levels of CD19 and CD10 positive cells at presentation or after treatment if the bone marrow was morphologically uninvolved. The upper limit of the CD20+range was 20% for patients (Figure 1) and 14% for normal subjects.

The objective of this study was to determine the normal range for CD20 positive cells in bone marrow, in the context of lymphoma and potential post-chemotherapy effects. This was established at a level higher than that previously suggested. A difference was demonstrated between levels of CD20 positive cells in infiltrated marrow and that of normal subjects and patients with no evidence of marrow infiltration.

Antigens defined by the CD19 and CD10 antibodies are expressed on cells appearing earlier in B-cell differentiation than CD20, and it might be expected that they would therefore be present in increased numbers following treatment if samples were analysed during active marrow recovery. This was not the case.

The range of CD20 positive cells was greater in patients with NHL than in normal subjects. A possible explanation for this is that the 'excess' CD20 positive cells are the phenotypic expression of a population of occult lymphoma cells which appear morphologically normal, the possibility of peripheral blood contamination being unlikely (Clarke *et al.*, 1986).

Assessment of the level of CD20 bearing cells is of importance when considering the efficacy of *in vitro* treatment with an antibody such as anti-B1 and complement. It has been demonstrated that, within the limits of flow cytofluorometric analysis, CD20 positive cells can be completely removed from bone marrow, which has a CD20 value in the range of 0–10%, by three cycles of treatment with anti-B1 and baby rabbit complement (Nadler *et al.*, 1981). These results have been confirmed in 38 patients with B-cell malignancy treated at St Bartholomew's Hospital (unpublished observations). However, immunophenotyping alone is not sensitive enough to detect very small populations of neoplastic cells. This is supported by the results of peripheral blood studies using molecular techniques in patients with lymphoma (Brada *et al.*, 1987).

Various methods have therefore been proposed to assess 'minimal residual disease' including dual fluorescence, analysis of specific chromosomal breakpoints (Weiss *et al.*, 1987), and more recently, molecular techniques using immunoglobulin and T-cell receptor gene rearrangements (Brada *et al.*, 1987). Such studies may help in the detection of morphologically undetectable lymphoma and further define the efficacy of *in vitro* techniques for removal of tumour cells from bone marrow being used for autologous transplantation.

We thank Coulter Immunology for providing both the monoclonal antibodies used in this study and a Coulter Epics C. We are very grateful to the 20 people who kindly donated bone marrow. Finally, we thank Mrs J. Newton and Elizabeth Hill for expertly typing this paper.

### References

ANDERSON, K.C., BATES, M.P., SLAUGHENHOUPT, B.L., PINKUS, G.S., SCHLOSSMAN, S.F. & NADLER, L.M. (1986). Expression of human B cell-associated antigens on leukaemias and lymphomas: a model of human B cell differentiation. *Blood*, **63**, 1424.

BAST, R.C. JR., DE FABRITIS, P., LIPTON, J. and 5 others (1985). Elimination of malignant clonogenic cells from human bone marrow using multiple monoclonal antibodies and complement. *Cançer Res.*, **45**, 499.

BENJAMIN, D., MAGRATH, I.T., DOUGLAS, E.C. & CORASH, L.M. (1983). Derivation of lymphoma cell lines from microscopically normal bone marrow in patients with undifferentiated lymphomas: evidence of occult bone marrow involvement. *Blood*, **61**, 1017.

BRADA, M., MIZUTANI, S., MOLGSAARD, H. and 4 others (1987). Circulating lymphoma cells in patients with B and T non Hodgkin's lymphoma detected by immunoglobulin and T-cell receptor gene rearrangement. *Br. J. Cancer*, **56**, 147.

CLARK, P., NORMANSELL, D.E., INNES, D.J. & HESS, C.E. (1986). Lymphocyte subsets in normal bone marrow. *Blood*, **67**, 1600.

NADLER, L.M., BOTNICK, L., FINBERG, R. and 5 others (1984). Anti-B1 monoclonal antibody and complement treatment in autologous bone marrow transplantation for relapsed B cell non Hodgkin's lymphoma. *Lancet*, **ii**, 427.

RITZ, J., SALLAN, S.E., BAST, R.C. JR. and 6 others (1982). Autologous bone marrow transplantation in CALLA positive acute lymphoblastic leukaemia after *in vitro* treatment with J5 monoclonal antibody and complement. *Lancet*, **ii**, 60.

ROHATINER, A.Z.S., BARNETT, M.J., ARNOTT, S. and 8 others (1986). Ablative therapy supported by autologous bone marrow transplantation (BMT) with *in vitro* treatment of marrow in patients with B cell malignancy. *Blood*, **68**, suppl. 1, 241a.

STASHENKO, P., NADLER, L.M., HARDY, R. & SCHLOSSMAN, S.F. (1980). Characterisation of a human B lymphocyte specific antigen. *J. Immunol.*, **125**, 1678.

STASHENKO, P., NADLER, L.M., HARDY, R. & SCHLOSSMAN, S.F. (1981). Expression of cell surface markers following human B lymphocyte activation. *Proc. Natl Acad. Sci. USA*, **78**, 3848.

WEISS, L.M., ROGER, M.D., WARNKE, R.A., SKLAR, J. & CLEARY, M.L. (1987). Molecular analysis of the t(14; 18) chromosomal translocation in malignant lymphomas. *N. Engl. J. Med.*, **317**, 1185.


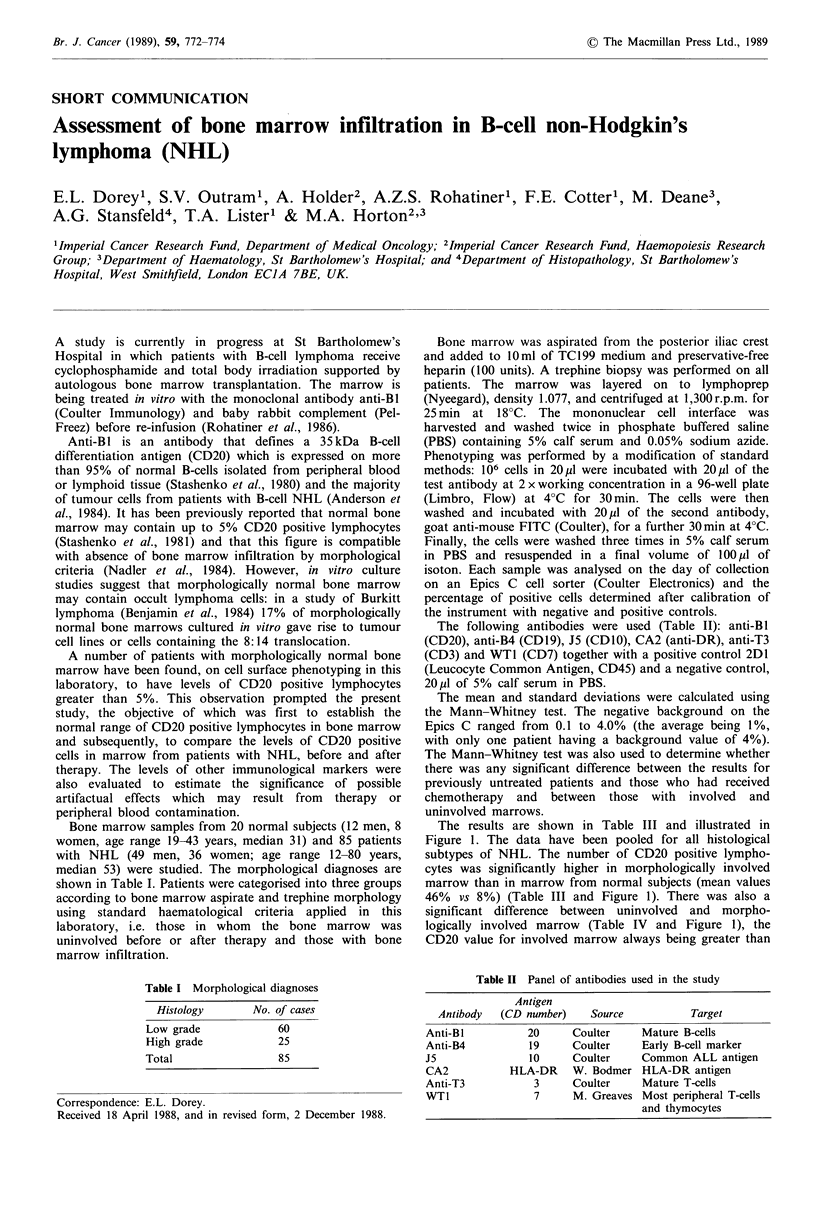

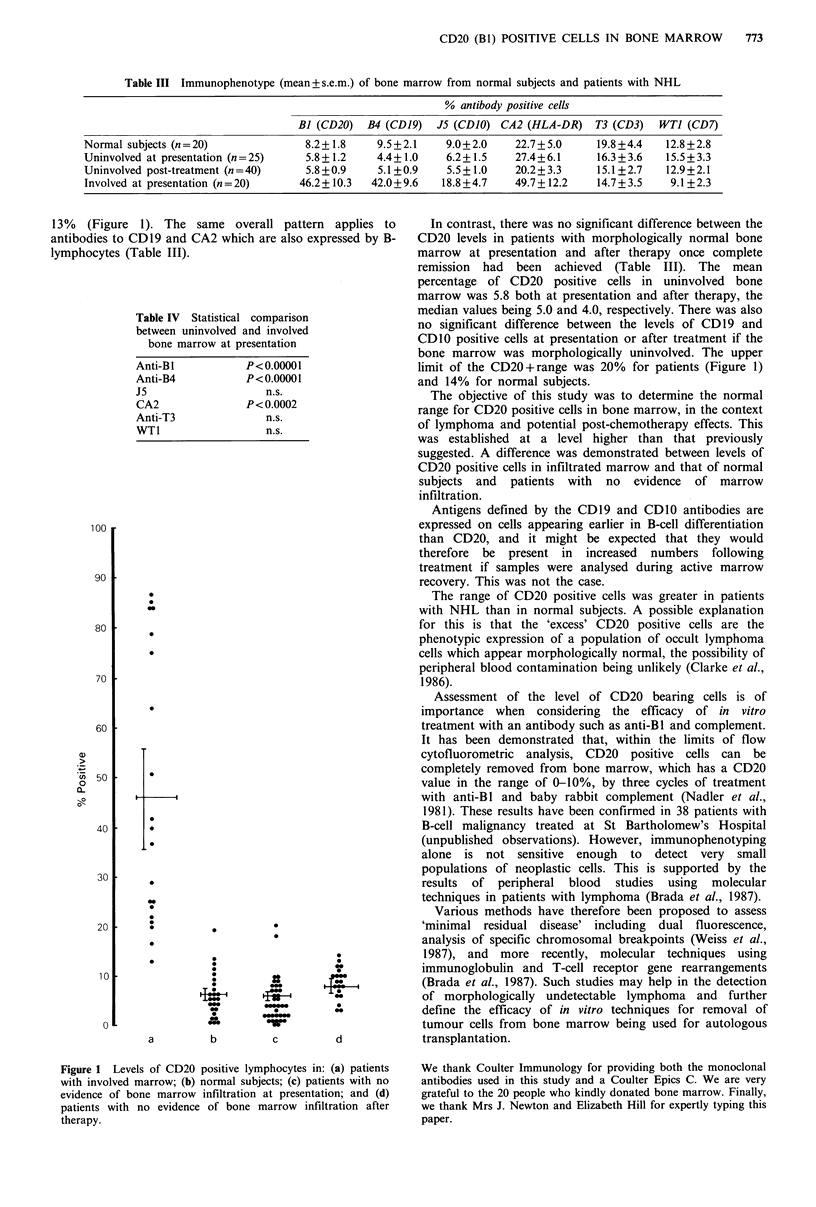

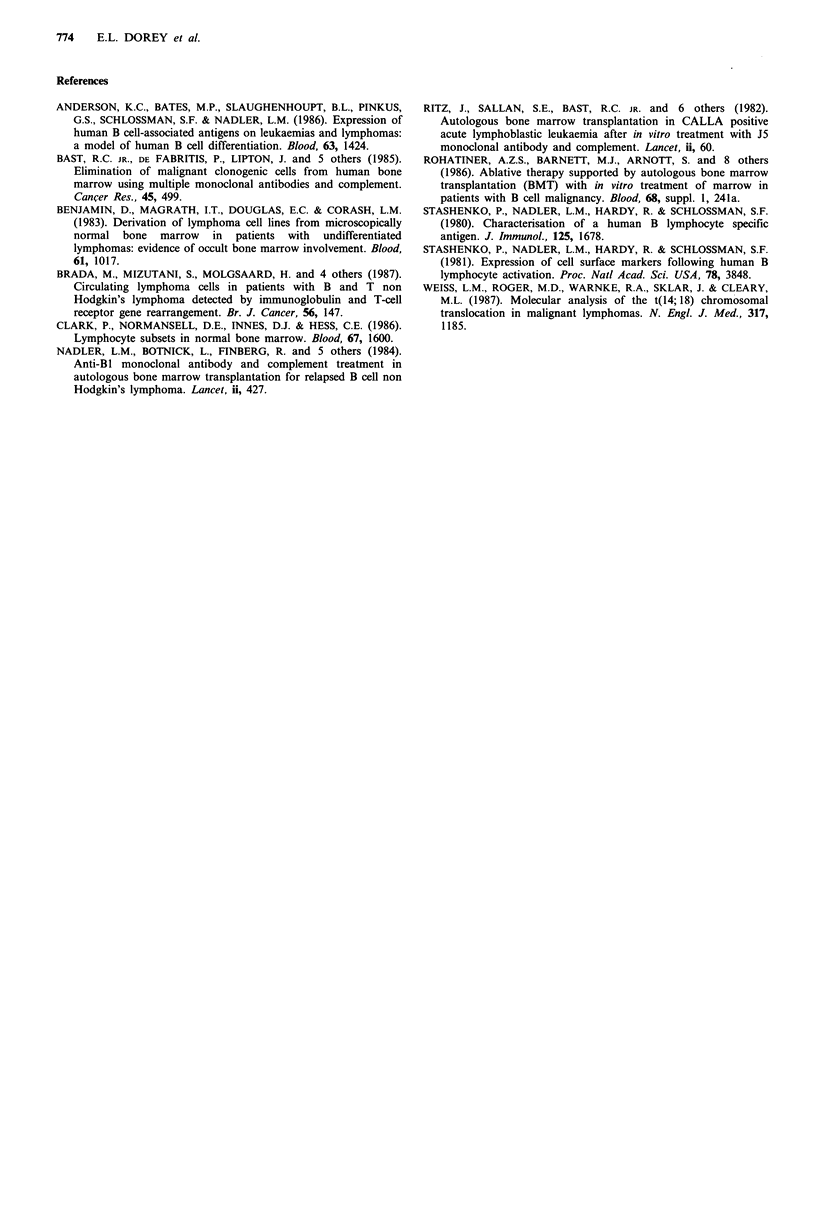


## References

[OCR_00319] Anderson K. C., Bates M. P., Slaughenhoupt B. L., Pinkus G. S., Schlossman S. F., Nadler L. M. (1984). Expression of human B cell-associated antigens on leukemias and lymphomas: a model of human B cell differentiation.. Blood.

[OCR_00325] Bast R. C., De Fabritiis P., Lipton J., Gelber R., Maver C., Nadler L., Sallan S., Ritz J. (1985). Elimination of malignant clonogenic cells from human bone marrow using multiple monoclonal antibodies and complement.. Cancer Res.

[OCR_00331] Benjamin D., Magrath I. T., Douglass E. C., Corash L. M. (1983). Derivation of lymphoma cell lines from microscopically normal bone marrow in patients with undifferentiated lymphomas: evidence of occult bone marrow involvement.. Blood.

[OCR_00338] Brada M., Mizutani S., Molgaard H., Sloane J. P., Treleaven J., Horwich A., Peckham M. J. (1987). Circulating lymphoma cells in patients with B & T non-Hodgkin's lymphoma detected by immunoglobulin and T-cell receptor gene rearrangement.. Br J Cancer.

[OCR_00344] Clark P., Normansell D. E., Innes D. J., Hess C. E. (1986). Lymphocyte subsets in normal bone marrow.. Blood.

[OCR_00348] Nadler L. M., Takvorian T., Botnick L., Bast R. C., Finberg R., Hellman S., Canellos G. P., Schlossman S. F. (1984). Anti-B1 monoclonal antibody and complement treatment in autologous bone-marrow transplantation for relapsed B-cell non-Hodgkin's lymphoma.. Lancet.

[OCR_00366] Stashenko P., Nadler L. M., Hardy R., Schlossman S. F. (1980). Characterization of a human B lymphocyte-specific antigen.. J Immunol.

[OCR_00371] Stashenko P., Nadler L. M., Hardy R., Schlossman S. F. (1981). Expression of cell surface markers after human B lymphocyte activation.. Proc Natl Acad Sci U S A.

[OCR_00376] Weiss L. M., Warnke R. A., Sklar J., Cleary M. L. (1987). Molecular analysis of the t(14;18) chromosomal translocation in malignant lymphomas.. N Engl J Med.

